# Factors associated with teenage marital pregnancy among Bangladeshi women

**DOI:** 10.1186/1742-4755-8-16

**Published:** 2011-05-20

**Authors:** Amir M Sayem, Abu Taher MS Nury

**Affiliations:** 1 Research Associate Bangladesh Institute of Social Research 6/14 (5th Floor), Block A Lalmatia, Dhaka-1207, Bangladesh; 2 Upazilla Family Planning Officer Narayanganj Sadar, Narayanganj Under Directorate General of Health Services Ministry of Health and Family Welfare Government of Bangladesh, Bangladesh

## Abstract

**Background:**

Teenage pregnancy is a public health concern both in developed and developing world. In Bangladesh, most of the first pregnancies occur immediately after marriage, especially among teenagers. Although women aged 15-29 years are the most fertility contributing women in Bangladesh, studies are not yet conducted on teenage pregnancy within this group of women. In the current study, an attempt had been made to identify the factors affecting teenage marital pregnancy in women aged 15-29 years.

**Methods:**

A cross sectional survey was carried out in 389 women, selected with a convenience sampling technique. Participants were selected on the basis of two criteria, such as married women and age within 15-29 years. We excluded women aged more than 29 years as we attempted to conduct study within high fertility contributing women and with the assumption that they may provide data subjected to relatively high level of recall bias as marital pregnancy may be a longer past event to them. In the analysis, we applied bi-variate and multi-variate logistic regression technique to find out odds ratio of teenage marital pregnancy.

**Results:**

Result revealed that 72.5% of the participants experienced first marital pregnancy during their teenage, with a mean age of 17.88 years (SD = 2.813). Multivariate logistic regression analysis revealed that participants aged 20-24 years had higher likelihood (OR 1.971, 95% CI 1.132 to 3.434), whereas participants aged 25-29 years had lower likelihood (OR 0.054, 95% CI 0.016 to 0.190) of experiencing teenage marital pregnancy compared to participants aged 15-19 years. In addition, participants desired for >2 children had significant higher odds (OR 3.573, 95% CI 1.910 to 6.684) and participants born in urban area had significant lower odds (OR 0.458, 95% CI 0.228 to 0.919) for teenage marital pregnancy.

**Conclusions:**

Based on the findings, we conclude that in order to reduce teenage marital pregnancy, consideration should be given on women's desired number of children and birth place so that women's desired number of children is limited to within two children, and that rural women get increased working and other related opportunities that may contribute in delaying teenage pregnancy.

## Background

Teenage pregnancy, pregnancy within 19 years of age, is a public health concern both in developed and developing counties [1,2]. Around the world, fifteen million women less than 20 years of age bear child which is one-fifth of all births [3], Evidence in developing world indicates that one-third to one-half of women become mothers within 19 years of age, making pregnancy related causes as leading causes of death [4]. Relatively, the situation in South Asian countries is severe as there are higher proportions of teenage pregnancies in this region due to common practice of early marriage and social expectation to have a child soon after marriage [5,6]. Evidence further indicates that nearly 60% of all girls are married by the age of 18 years and one fourth is married by the age of 15 years in South Asia [7], whereas within South Asia, the recorded teenage pregnancy rate is highest in Bangladesh (35%) followed by Nepal (21%) and India (21%) [8].

Data show that globally 529,000 women die in every year due to pregnancy and child birth related complications [3], whereas the risk of death due to pregnancy-related causes is double among women aged 15-19 compared to women in their twenties [9]. This may be due to several reasons including low use of prenatal care in the first trimester [10] and insufficient physical development to bear child. In addition, teenage pregnancy can significantly affect socio economic situations of women and family, including maternal education, women's employment opportunities, marital stability, and increased economic and social dependency on family and neighbors [11]. As a result of low educational and occupational attainment, and low socioeconomic status, adolescent pregnancy contributes to the perpetuation of the poverty cycle [12,13], making already vulnerable family to more vulnerable.

Recently, reviews have been conducted on teenage pregnancies in developing countries [14] but no focus has been made specifically in Bangladesh context. More importantly, information related to risk factors affecting teenage pregnancy is absent in developing country context, including Bangladesh, although most of the marriages occurs before 18 years of legal age at marriage in this country [15]. Under these circumstances, in the current study, an attempt has been made to identify the factors affecting teenage marital pregnancy in Bangladesh context. We considered women aged 15-29 years because they are the most fertility contributing women in Bangladesh, whereas women aged >29 yeas have much lower fertility rates [15]. We considered marital pregnancy because three is no official record of pregnancy of unmarried women in Bangladesh. In addition, women may neither become mother prior to marriage nor reveal such highly sensitive information in Bangladesh context, even if women become mother prior to marriage.

## Methods

### Participants

We carried out this study in 389 married women, selected with a convenience sampling technique, from both rural and urban area. The participants of this study consisted of 57.7% of the total participants of a larger survey, carried out among 674 married women aged 15-49 years in order to investigate health care exclusion in both urban and rural area. We excluded 42.3% participants because they are the most fertility contributing women in Bangladesh, whereas women aged >29 yeas have much lower fertility rates [15] and because inclusion of women aged >29 years could produce relatively higher level of recall bias due to relative longer past event.

In the current study, more than one fourth participants (26.2%) had 15-19 years of age, whereas 30.8% and 42.9% had respectively 20-24 and 25-29 years of age (not shown). Majority of the participants had education (85.6%) which is almost similar to husband's education (86.6%). Majority of the participants (92.0%) were housewife while only 8% participants were working. More than four fifth of the participants' birth place was rural (81.2%), while birth place was urban for 18.8% participants. Mostly, participants had nuclear family consisted of 71.2% against joint family consisted of 28.8%. One sixth participants had no exposure to mass media, whereas 49.4% and 34.2% had exposure to one and more than one mass media respectively. Almost equal number of participants desired for 2 (49.6%) and >2 (50.4%) children.

### Procedure

The survey was carried out with a pilot tested semi-structured interview questionnaire. With the assistance of six female trained interviewers, we collected necessary information from the participants. All the interviewers were 18-22 years old within 12 to 16 years of schooling. In addition, interviewers had experience in data collection, which facilitated interviews in the current study. Interviewers reached to potential participants' home with a convenience approach and asked several questions on different issues including background characteristics and pregnancy in their teenage (10-19 years). Prior to interview, interviewers took informed consent from all the participants. Interviews were carried out in July, 2010; on an average, each interview lasted for 25 minutes.

### Measuring variables

Dependent variable, teenage marital pregnancy, was measured in completed years of pregnancy within the age of 10-19 years. Participants who were pregnant within 19 years were categorized as 1 (yes) while no pregnancy within 19 years was categorized as 0 (no). To find out the factors affecting teenage marital pregnancy, we considered eight explanatory variables, such as participants' age at present (0 = 15-19, 1 = 20-24 and 2 = 25-29), education (0 = no and 1 = yes), occupation (0 = not working and 1 = working), desired number of children (0 = 2 and 1=>2), husband's education (0 = no and 1 = yes), birth place (0 = rural and 1 = urban), family type (0 = nuclear and 1 = joint) and mass media exposure (0 = no exposure, 1 = exposure to 1 media and 2 = exposure to2 media).

To understand participants' education, interviewers asked whether they attended any formal school. Participants responded positive were considered as having education, while negative as having no education. With regard to participants' occupation, interviewers asked them about their type of work. Response categories were 0 = housewife, 1 = government service, 2 = private service and 9 = others. As frequency for different working categories except housewife was very low, we merged all the working categories into a single category, i.e., working, whereas housewives were considered as not working. Similar procedure to that of participants' education was applied to know the status of husband's education. To know participants' family type, interviewers asked them whether they belong to nuclear or join family. By the joint family we mean a family where marred women were living with other family members of husband, especially father, mother, brother and sister for the last one year. In contrast, nuclear family means a family where women were living with her husband and her children for the same period. With reference to mass media exposure to participants, interviewers asked whether participants had exposure to media. Participants' responses varied from no medium, 1 medium and more than one media. Mostly participants had exposure to television (80.2%), followed by radio (25.4%) and newspaper (16.7%).

### Data analysis

With necessary editing and coding, we entered data into computer using SPSS version 11.5. Necessary screening was performed subsequently through frequency analysis to check data consistency and missing data. Although initial entry resulted in some missing data entry, we entered missing data from the questionnaire following the serial number assigned, prior to data entry, to each of the collected questionnaire. At the analysis stage, we distributed frequency across different characteristics including dependent variable. We also performed bi-variate analysis to identify the associations of teenage marital pregnancy (Table 1). In this regard, we considered logistic regression analysis as the dependent and independent variables were categorical. However, we did not limit out analysis to bi-variate associations, since bi-variate analysis only tells the relationship between two variables, which is often considered as bias relation due to influence of other variables that is unobserved in bi-variate associations. To identify the independent impact of independent variables, we performed multi-variate logistic regression analysis. All the variables used in the bi-variate analysis, were used in the multi-variate analysis. In the multi-variate analysis, we used enter method. Results of multi-variate logistic analysis are presented in Table 2.

**Table 1:** Bi-variate logistic regression analysis of teenage marital pregnancy

				**95.0% C.I**.	
				
	B	**S.E**.	OR	Lower	Upper
*Age*					
15-19 ^RC^	1.000		1.000		
20-24	0.344	0.252	1.410	0.860	2.311
25-29	-2.727	0.618	0.065***	0.019	0.220
*Education*					
No ^RC^	1.000		1.000		
Yes	-1.289	0.448	0.276**	0.115	0.664
*Occupation*					
Not working ^RC^	1.000		1.000		
Working	-0.082	0.413	0.921	0.410	2.070
*Desire number of children*					
2 ^RC^	1.000		1.000		
>2	1.064	0.240	2.899***	1.811	4.640
*Husband education*					
No ^RC^			1.000		
Yes	-0.998	0.423	0.369*	0.161	0.845
*Birth place*					
Rural ^RC^			1.000		
Urban	-1.358	0.271	0.257***	0.151	0.438
*Family type*					
Nuclear ^RC^			1.000		
Joint	-0.136	0.248	0.873	0.537	1.418
*Mass Media exposure*					
No exposure ^RC^			1.000		
Exposure to 1 media	-1.275	0.387	0.280**	0.131	0.597
Exposure to >1 media	-0.801	0.247	0.449**	0.277	0.728

B = Beta Coefficient; SE = Standard Error, OR = Odds Ratio; CI = Confidence Interval

^RC ^= Reference category

***p < 0.001, **p > 0.01, *p < 0.05

**Table 2:** Multivariate logistic regression analysis of teenage marital pregnancy

	B	**S.E**.	OR	**95.0% C.I**.	
				
				Lower	Upper
*Age*					
15-19 ^RC^			1.000		
20-24	0.679	0.283	1.971*	1.132	3.434
25-29	-2.912	0.639	0.054***	0.016	0.190
*Education*					
No ^RC^			1.000		
Yes	-0.841	0.515	0.431	0.157	1.184
*Occupation*					
Not working ^RC^			1.000		
Working	0.763	0.472	2.144	0.850	5.412
*Desire number of children*					
2 ^RC^			1.000		
>2	1.274	0.320	3.573***	1.910	6.684
*Husband education*					
No ^RC^			1.000		
Yes	-0.313	0.526	0.731	0.261	2.051
*Birth place*					
Rural ^RC^			1.000		
Urban	-0.782	0.355	0.458*	0.228	0.919
*Family type*					
Nuclear ^RC^			1.000		
Joint	-0.272	0.311	0.762	0.414	1.402
*Mass media exposure*					
No exposure ^RC^			1.000		
Exposure to 1 media	-0.212	0.476	0.809	0.318	2.056
Exposure to >1 media	-0.328	0.310	0.721	0.392	1.324

B = Beta Coefficient; SE = Standard Error, OR = Odds Ratio; CI = Confidence Interval

^RC ^= Reference category

***p < 0.001; **p > 0.01, *p < 0.05

## Results

### Adolescent marital pregnancy

Around three fifth participants (72.5%) aged 15-29 years experienced marital pregnancy within (not shown), with a mean age of 17.88 years (SD = 2.813). Bi-variate logistic regression analysis revealed that participants' teenage pregnancy varied by different characteristics (Table 1). Participants aged 20-24 years had higher likelihood for teenage marital pregnancy (OR 1.410, 95% CI 0.860 to 2.311) but result was not statistically significant, whereas participants aged 25-29 years had significant lower likelihood of teenage pregnancy (OR = 0.065, 95% CI 0.019 to 0.220). Educated participants had significant lower odds ratio of teenage marital pregnancy (OR 0.276, 95% CI 0.115 to 0.664) compared to their uneducated counterparts. Although working participants were less likely to experience teenage marital pregnancy, result was not statistically significant. Participants desired for more than two children were more likely to experience teenage marital pregnancy (OR 2.899, 95% CI 1.811 to 4.640) compared to participants desired for 2 children. Participants with educated husband were significantly less likely to experience teenage marital pregnancy (OR 0.369, 95% CI 0161 to 0.845). Participants born in urban area had lower tendency to experience teenage marital pregnancy (OR 0.257, 95% CI 0.151 to 0.438). Similarly, participants exposed to one, and more than one mass media were less likely to experience teenage marital pregnancy (OR 0.280, 95% CI 0.131 to 0.597 and 0R 0.449, 95% CI 0.277 to 0.728 respectively).

### Multivariate associations of adolescent pregnancy

Multivariate logistic regression analysis was performed to identify the factors affecting teenage marital pregnancy (Table 2). Similar to bi-variate result, participants aged 20-24 years had higher likelihood (OR 1.971, 95% CI 1.132 to 3.434) while participants aged 25-29 years had lower likelihood (OR 0.054, 95% CI 0.016 to 0.190) of teenage marital pregnancy compared to participants aged 15-19 years of age. Similar to that of bi-variate result, participants desired for >2 children were significantly more likely to experience teenage marital pregnancy compared to participant desired for 2 children (OR 3.573, 95% CI 1.910 to 6.684). Participants born in urban area were also significantly less likely to experience teenage marital pregnancy (OR 0.458, 95% CI 0.228 to 0.919), which is also similar to that in bi-variate analysis. No more variables appeared to have significant impact on teenage marital pregnancy in multivariate logistic regression analysis.

## Discussion

This study found that majority of the participants had experienced teenage marital pregnancy. Most of the pregnancies occurred at the age of 15-19 years, which is consistent with another study conducted in rural areas of Bangladesh, where mean age at first birth was relatively lower [16]. Teenage pregnancy was also reportedly higher in USA within this age group, i.e., 94 pregnancies per 1000 teens [17]. The mean age of teenage pregnancy among the high fertility contributing women was 17.88 years, which is almost similar to another study conducted in Equador where reported mean age was 16.5 years [18]. The reported mean age at marital pregnancy, in the current study, indicates that still many of the marriages occur at less than 18 years of minimum legal age which may contribute to high fertility in the future as early marital pregnancy may cause a longer reproductive life among these women, provided women are fecund and do not use family planning method.

We further investigated the factors affecting teenage marital pregnancy. As we found, three variables affected teenage pregnancy. Of the significant predictors, in our study, age appeared to have a mixed effect on teenage marital pregnancy, i.e., women aged 20-24 years were more likely while women aged 25-29 years were less likely to experience teenage marital pregnancy. This is rather unexpected finding as contraceptive prevalence rate (CPR) was higher five years ago [[19] NIPORT et al, 2004]. compared to the current CPR [[15] NIPORT et al, 2007] suggesting that women aged 20-24 years should have lower teen pregnancy due to higher CPR at that time. Perhaps this unexpected finding may be because of higher traditional contraceptive method use in five years ago compared to current situation where despite decreased CPR modern method relatively increased [[15] NIPORT et al, 2007]. It is, thus, expected that due to the use of traditional method, there was high failure rate, resulting in unintentional pregnancy among women aged 20-24 years who were within teenagers before five years.

Consistent with other studies [1,20], this study identified that women's education had negative association with teen marital pregnancy in bi-variate analysis. Insignificant result in multi-variate analysis may be indicative of more importance of other factors and incapacity of education to influence adolescent pregnancy in multi-dimensional settings. Similarly, mass media exposure appeared to have significant negative impact on teenage marital pregnancy in bi-variate analysis but not in multivariate analysis. Such unexpected negative impact of mass media on teenage marital pregnancy may be indicative of a great concern, as awareness is mainly raised through mass media campaign. Perhaps, women might have known different issues, through mass media, such as information about pregnancy care and breastfeeding, but not disadvantages of early or teenage pregnancy. Such information along with pressure from other family members to prove them as fertile might have forced women to bear child immediately after marriage.

The current study identified that women desired for more than two children were more likely to experience teenage marital pregnancy. Another study indicates that women desired for more children less likely to use contraceptive [21] and hence more likely to go for frequent childbearing. Our study further revealed that majority of the women desired for >2 children which is similar to another study conducted in rural areas of Bangladesh [21] but higher than that of national data where ideal number of children was within two for 74.4% women [15]. The difference, between national data and the current study, may be because of the difference in study population, i.e., national data were collected from women aged 15-49 years while our data were collected from women aged 15-29 years. Perhaps a vast proportion of women (around one third) aged >29 years could understand the socio-economic and other related problems in higher number of child birth, since most of them already taken two or more children, resulted in lower ideal number of children for high proportion of women (2 children). On the other hand, as women aged 15-29 years are women who contribute higher fertility compared to women of more than 29 years, it is expected that desire number of children would be higher in our study.

Finding further indicated that women born in urban area were less likely to experience teenage pregnancy compared to their rural counterparts. It is expected because age at first marriage is lower for rural women than their urban counterparts in Bangladesh [15]. In addition, relative substantial cultural stimulation of early child bearing and women's lower working opportunities and hence women's more concentration in early establishment of family might have important influence for women born in rural areas. Conversely, urban women might have chance to avoid social pressure through work (in this study, 5.7% women born in urban area were working against 2.3% in rural area) and the related advantages, that come with urban culture, and hence less concentration in early family establishment along with higher age at married might contributed lower teenage pregnancy. If this is the case, cultural transition or urbanization remains as the main alternative for lessening teenage marital pregnancy in rural women in Bangladesh.

Although our study provided some important issues of teenage marital pregnancy, findings of this study should be considered in the light of methodological limitations. We conducted this study on the basis of convenience sampling technique. Although it is inexpensive way for ensuring sufficient number of participants in a study, findings can be highly unrepresentative [22]. Therefore, caution should be taken to generalize findings of this study beyond young women and the sample women. Furthermore, our further investigation revealed that only there variables appeared significant in the multi-variate analysis. It is, thus, possible that there are other important factors that influence teenage pregnancy, suggesting a more in-dept future study considering broader socio-economic context for better understanding factors affecting teenage marital pregnancy.

## Conclusions and Recommendations

Our study found that majority of the women aged 15-29 years had experienced teenage marital pregnancy, indicating a serious public health concern. Current study also identified several factors, contributed to the higher teenage pregnancy, such as women's age, desire number of children and birth place. Based on the findings of the present study, we recommend that intervention should be given to the reduction of desired number of children. As this study indicated that still half of the women desired for more than two children. Higher desired number of children leads women to have children immediately after marriage; more importantly, as desired number children is more of cultural, there should a long term strategy to tackle this issue culturally. Community based campaign, since mass media exposure did not appear significant in multi-dimensional social settings, for limiting desired number of children within two may be considered. In this regard, disadvantages of higher desired number of children may be the content. However, prior to developing content of the campaign, formative research would be necessary to provide culture specific information so that they are culturally accepted to women. In addition, special emphasis should be given among women born in rural areas where relatively awareness is very low along with absence of other civic opportunities that come in urban areas.

## Competing interests

The authors declare that they have no competing interests.

### Financial competing interests

1. No organization financed this study. This is the study carried out by the author's own funds

2. No organization is likely to loose financially

3. No reimbursements, fees, funding received from any organization relating to patents of the content of the manuscript

4. No, we have no financial competing interest

### Non-financial competing interests

No, we do not have political, personal, religious, academic, ideological, intellectual, commercial or any other competing interest.

## Authors' contributions

AMS (Amir Mohammad Sayem) developed concept, participated in questionnaire development, data analysis, drafted literature review, drafted discussion and prepared final version of the manuscript. ATMSN (Abu Taher Md. Sanaullah Nury) participated in concept development, questionnaire development, participated in data analysis, drafted result section, and participated in discussion section and commented on all the section prior to finalization of the manuscript. Both authors read and approved the final manuscript.
